# Telephone-Based Shared Decision-making for Lung Cancer Screening in Primary Care

**DOI:** 10.1007/s13187-019-01528-z

**Published:** 2019-05-09

**Authors:** Heather Bittner Fagan, Nicole A. Fournakis, Claudine Jurkovitz, Anett M. Petrich, Zugui Zhang, Nora Katurakes, Ronald E. Myers

**Affiliations:** 1 Department of Family and Community Medicine, Christiana Care Health System, Wilmington, DE USA; 2 Office of Health Equity, Christiana Care Health System, Wilmington, DE USA; 3 Department of Family Medicine, Wilmington Annex Office 328, 1400 Washington St., Wilmington, DE 19801 USA; 4 Value Institute, Christiana Care Health System, Edgemoor, DE USA; 5 Division of Population Science, Department of Medical Oncology, Sidney Kimmel Medical College, Thomas Jefferson University, Philadelphia, PA USA; 6 Community Health Outreach and Education, Helen F. Graham Cancer Center, Christiana Care Health System, Edgemoor, DE USA

**Keywords:** Shared decision-making, Lung cancer screening, Primary care, Telephone-based intervention

## Abstract

The national rate of  lung cancer screening, approximately 3–5%, is too low and strategies which include shared decision-making and increase screening are needed. A feasibility study in one large primary care practice of telephone-based delivery of decision support via an online tool, the Decision Counseling Program© (DCP) was administered to patients eligible for lung cancer screening according to USPSTF screening guidelines. We collected data on demographics, decisional conflict, and conducted chart audits to ascertain screening. From electronic medical record data, we identified 829 age-eligible current or former smokers. Of the 297 individuals reached, 54 were eligible and 28 were recruited to the study and 20 underwent the DCP© intervention. Participants in the intervention were more likely to complete low-dose CT scans at 90 days. Current smokers were less likely to complete the DCP. Women were less likely to complete LDCT. This non-persuasive, high-quality shared decision-making intervention significantly increased lung cancer screening and was feasible in real-world clinical care. This intervention offers a promising model whereby patients can be supported in a decision, based on their values and beliefs while also supporting gains in lung cancer screening.

## Introduction

Lung cancer is the leading cancer killer in the USA. Each year, more people die from lung cancer than from colon, breast, and prostate cancers combined. Results of the National Lung Cancer Screening Trial (NLST) showed that annual lung cancer screening with a low-dose computed tomography (LDCT) can find lung cancer at an earlier stage and reduce the risk of dying from this disease. The NLST was a randomized trial of LDCT versus chest x-ray in individuals at high risk for lung cancer [1]. The NLST demonstrated a 20% reduction in mortality in groups that carried out LDCT screening.

In 2013, based on the NLST, the United States Preventive Services Task Force (USPSTF) recommended annual use of LDCT scans to screen for lung cancer in high-risk individuals. High-risk individuals are defined as persons between the ages of 55 and 80 years of age, with at least a 30 pack-year history of smoking, who either currently smoke or quit less than 15 years ago (level B recommendation) [2]. In response to the USPSTF recommendation, the Center for Medicare and Medicaid Services (CMS) included lung cancer screening (LCS) as a covered benefit. But, importantly, CMS requires that “a beneficiary must receive a written order for LDCT lung cancer screening during a lung cancer screening counseling and shared decision making (SDM) visit.”

Currently, lung cancer screening rates in the USA are very low—just 3.3% in 2010 and 3.9% in 2015 [3]. Not yet known is how to effectively promote and increase lung cancer screening, while supporting patients and physicians in this high-stakes decision. All screening tests are associated with the risk of a false alarm, unnecessary treatment, and incidental findings. Lung cancer screening is a high-stakes decision, because the potential mortality benefit of LCS is high but is not yet demonstrated in real-world clinical care. This benefit should be considered along with the risk of having incidental findings, which may lead to invasive diagnostic procedures, e.g., bronchoscopy and even thoracic surgery.

SDM is a process by which a health care provider and patient make a health care decision together based on the values and beliefs of the patient and incorporating the scientific evidence [4]. However, little is known about how SDM should be implemented in practice. Recent evidence suggests that SDM for lung cancer screening may be done poorly in clinical practice [5]. Traditionally, emphasis has been placed on the doctor-patient dyad [6], and SDM takes place in a patient-provider interaction within the context of the scheduled face-to-face doctor-patient visit. In part due to practical considerations such as time constraints, there is rising interest in facilitating SDM outside of the usual doctor-patient encounter and in utilizing other health professionals in order to best prepare and assist the patient in their decision-making process [4]. This study tested the feasibility of a telephone-delivered, primary care–integrated SDM intervention for LDCT.

## Methods

This study was approved by the Christiana Care Health System (CCHS) Institutional Review Board. This was a pilot study conceived to test the feasibility of delivering this novel intervention in hand with an active primary care practice.

### Setting

CCHS is an independent academic health care system and the predominant health care provider in Delaware. Delaware demographics closely resemble the demographic distribution of the USA in terms of race and gender. The primary care practice was chosen because of large patient volume and because it included two sites, one urban and one suburban.

### Eligibility and Participant Recruitment

Study participant eligibility criteria were based on the USPSTF recommendations for lung cancer screening. To be eligible, the patient had to be between the age of 55 and 80 and be a current or former smoker with at least a 30 pack-year history, has not quit smoking for more than 15 years, and has not have received a LDCT scan within the last year.

Using practice-based electronic medical record(EMR) data, a data analyst generated a list of potentially eligible patients: current and former smokers between the age of 55 and 74 and included provided demographic and contact information. Patient lists were uploaded into a Research Electronic Data Capture (REDCap) database under a randomly assigned record ID. Charts were reviewed manually in the EMR and any patient who had a CT scan of the lungs in the past year was eliminated. Patient lists were sent to the patient’s primary care physicians for review. Primary care physicians were invited to exclude patients from being contacted for enrollment into the study for any reason, including excess comorbidities or psychological conditions.

Up to three initial telephone call attempts to contact the patient were made. Once a patient was contacted, eligibility was confirmed by self-report. Patients who met the eligibility criteria were consented and enrolled into the study. All current smokers, regardless of eligibility, were offered a direct referral to the Delaware Quitline, a toll-free tobacco cessation hotline that provides tobacco users the option to receive counseling by phone or in person.

### Decisional Conflict Survey

During the initial call after consent and enrollment, a baseline survey was administered. The 10–15-min baseline survey included a 16-item decisional conflict scale measuring the patient’s perceptions of the decision-making process around lung cancer screening. Using the standard 5-point Likert scale (0 = “strongly agree” to 4 = “strongly disagree”), the decisional conflict scale included a mix of statements that measured the following constructs: feeling uncertainty, uninformed, unsupported, and unclear [7]. Selected responses were recorded in REDCap. Constructs were scored according to the Cochrane systematic review of trials of patient decision aids [8] where statements are broken into the construct subscores, summed, divided by 3 then multiplied by 25 to convert to a 0–100 scale. After completion of the baseline survey, the patient was scheduled for a telephone-based SDM session with a trained decision counselor. At the conclusion of the call, all participants were mailed a copy of the consent form and educational materials about the risks and benefits of lung cancer screening.

### Intervention

The decision counselor called each patient at the appointed time. During the phone-based appointment, the decision counselor reviewed the educational materials that were mailed and guided the patient through decision counseling session using an online software application, the Decision Counseling Program© (DCP). The DCP is used to clarify preference for LDCT screening and identify factors explaining preference. The DCP is not persuasive. More specifically, the decision counselor asked the patient to identify factors that would influence his/her decision to screen or not to screen for lung cancer. The decision counselor entered each elicited factor from the patient into the online program. The decision counselor then reviewed the factors with the patient and asked the patient to select up to three factors (between both options) that would most likely influence their screening decision. Next, the decision counselor asked the patient to indicate the importance of each factor, as well as determine the relative importance of factor pairs. At the conclusion of the session, the decision counselor used the DCP to generate a summary report that included information on the patient’s preference: prefer to screen, uncertain about screening, or prefer not to screen. The decision counselor shared the result with the patient and asked for his/her self-reported screening decision. If the patient reported s/he did not want to screen or were uncertain, the decision counselor recommended the patient speak with their primary care physician to discuss screening. If the patient reported s/he wanted to screen, the decision counselor referred the patient directly to the Christiana Care comprehensive lung health screening program (LHSP).

After the patient completed the DCP intervention and within 30 days from initial contact, the research assistant followed up with the enrolled patient by phone to conduct the post decisional conflict survey. After 90 days from initial contact, the research assistant conducted chart audits of enrolled participants to verify if a LDCT screening appointment was made and if the patient actually screened.

## Data Collection and Statistical Analysis

Continuous variables (pack-years and decisional conflict scores) were summarized using means and standard deviations (SD) and compared using non-parametric Wilcoxon rank sum tests. Categorical variables (gender, age, race, ethnicity, insurance, employment, marital status, smoking status, and DCP completion) were reported using frequencies and percentages, and compared with the Pearson chi-square test or Fisher exact test when necessary. *P* < 0.05 was the threshold for statistical significance. All analyses were performed using SAS 9.4 (SAS Institute Inc., Cary, NC). For patients who completed both the baseline and the follow-up decisional conflict scale, the mean differences in each domain and total score of the decisional conflict scale were calculated.

## Results

### Study Participation

**Fig. 1:**
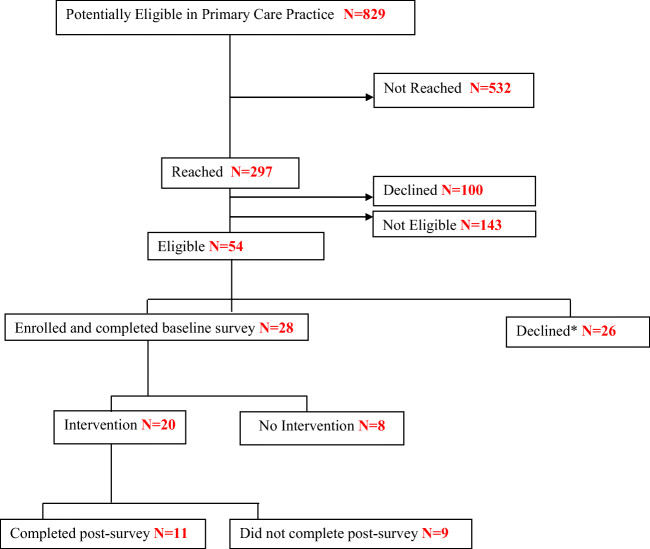
Patient recruitment flow chart. *Before completion of baseline survey

Figure 1 shows the consolidated chart of patient recruitment. A total of 829 potentially eligible patients were identified and 532 could not be contacted by telephone. From this remaining pool, 297 were reached by phone, and 54 of these individuals were determined to be eligible for lung cancer screening. Of the 54 eligible participants, 28 (57%) were enrolled in the study.

Table 1 shows the characteristics of the study population. Among the 28 participants, about 54% were female and 18% were African American and 89% were non-Hispanic. Most were age 55–64 years (64%) with a mean age of 63, publicly insured (61%), unemployed or retired (61%), and single/divorced/widowed (71%). Of the 28, 19 were current smokers and 9 were former smokers. Twenty participants completed a DCP session and 9 completed LDCT screening.

**Table 1:** Patient factors and LDCT screening at 90 days

	All (*N* = 28)	Screen (*N* = 9)	Not Screen (*N* = 19)	
Variables	*N* (%)	*N* (%)	*N* (%)	*p* value
Gender				
Male	13 (46.4)	7 (77.8)	6 (31.6)	0.04
Female	15 (53.6)	2 (22.2)	13 (68.4)	
Age (years) mean = 62.64				
55–64	18 (64.3)	6 (66.7)	12 (63.2)	0.47
≥ 65	10 (35.7)	3 (33.3)	7 (36.8)	
Race				
White	22 (78.6)	6 (66.7)	16 (84.2)	0.35
African American	5 (17.9)	3 (33.3)	2 (10.5)	
Other	1 (3.6)	0 (0.0)	1 (5.3)	
Ethnicity				
Hispanic	1 (3.6)	1 (11.1)	0 (0.0)	0.32
Non-Hispanic	25 (89.3)	8 (88.9)	17 (89.5)	
Missing	2 (7.1)	0 (0.0)	2 (10.5)	
Insurance				
Private	10 (35.7)	3 (33.3)	7 (36.8)	0.75
Public	17 (60.7)	6 (66.7)	11 (57.9)	
Uninsured	1 (3.6)	0 (0.0)	1 (5.3)	
Employment status				
Employed	6 (21.4)	4 (44.4)	2 (10.5)	0.16
Unemployed/retired	17 (60.7)	5 (55.6)	12 (63.2)	
Missing	5 (17.9)	0 (0.0)	5 (26.3)	
Marital status				
Married	7 (25.0)	2 (22.2)	5 (26.3)	0.94
Single/divorced/widow	20 (71.4)	6 (66.7)	14 (73.7)	
Missing	1 (3.6)	1 (11.1)	0 (0.0)	
Smoking status				
Current smokers	19 (67.9)	6 (66.7)	13 (68.4)	0.93
Former smokers	9 (32.1)	3 (33.3)	6 (31.6)	
DCP completion				
Complete DCP	20 (71.4)	9 (100.0)	11 (57.9)	0.03
Incomplete DCP	8 (28.6)	0 (0.0)	8 (42.1)	
Pack-years mean (SD) median	46.54 (18.91) 42.0	55.00 (27.5) 42.0	42.97 (13.3) 48.0	0.27
Baseline decisional conflict	Mean (SD)	Mean (SD)	Mean (SD)	*p* value
Uncertain	32.74 (17.3)	26.8 (5.6)	35.53 (20.2)	0.10
Uninformed	28.57 (17.0)	29.6 (18.7)	28.07 (16.7)	0.83
Unclear	29.46 (15.6)	28.7 (8.4)	29.83 (18.3)	0.86
Unsupported	25.00 (13.2)	23.1 (3.7)	25.88 (15.9)	0.62
Effective decision	27.90 (12.1)	25.0 (3.1)	29.28 (14.4)	0.39
Total score	28.68 (11.9)	26.6 (3.6)	29.69 (14.3)	0.53

Table 1 also shows the patient characteristics and factors associated with LDCT at 90 days post-intervention.

Males were significantly more likely to complete LDCT (*p* = 0.04). DCP completion was positively and significantly associated with LDCT (*p* = 0.03). Although not significant, the number of pack-years appears higher in patients who screened than in patients who did not. Also not statistically significant, a higher level of baseline uncertainly appears to be negatively associated with LDCT (*p* = 0.1).

Table 2 shows factors associated with DCP completion. Only smoking status was significantly associated with DCP completion with current smokers being less likely to complete the DCP (*p* = 0.02). Other factors including gender, age, race, ethnicity, marital status, insurance, employment, and decisional conflict did not demonstrate a statistically significant association with DCP completion.

**Table 2:** DCP completion according to selected variables

	Complete DCP (*N* = 20)	Incomplete DCP (*N* = 8)	
Demographic variables	*N* (%)	*N* (%)	*p* value
Gender			
Male	11 (55.0)	2 (25.0)	0.22
Female	9 (45.0)	6 (75.0)	
Age (years)			
55–64	12 (60.0)	5 (62.5)	0.81
≥ 65	8 (40.0)	3 (37.5)	
Race			
White	15 (75.0)	7 (87.5)	0.64
African American	4 (20.0)	1 (12.5)	
Other	1 (5.0)	0 (0)	
Ethnicity			
Hispanic	1 (5.3)	0 (0.0)	0.52
Non-Hispanic	16 (84.2)	9 (100.0)	
Missing	2 (10.5)	0 (0.0)	
Insurance			
Private	7 (35.0)	3 (37.5)	0.81
Public	12 (60.0)	5 (62.5)	
Uninsured	1 (5.0)	0 (0.0)	
Employment status			
Employed	5 (25.0)	1 (12.5)	0.54
Unemployed/retired	12 (60.0)	5 (62.5)	
Missing	3 (15.0)	2 (25.0)	
Marital status			
Married	6 (30.0)	1 (12.5)	0.63
Single/divorced/widow	13 (65.0)	7 (87.5)	
Missing	1 (5.0)	0 (0.0)	
Smoking status			
Current smokers	11 (55.0)	8 (100.0)	0.02
Former smokers	9 (45.0)	0 (0.0)	
Pack-years	Mean (SD)	Mean (SD)	*p* value
	49.7 (21.4)	39.1 (7.9)	0.19
Baseline decisional conflict	Mean (SD)	Mean (SD)	*p* value
Uncertain	33.7 (17.2)	30.2 (18.3)	0.64
Uninformed	27.5 (16.9)	31.2 (18.2)	0.60
Unclear	18.7 (13.4)	31.2 (21.2)	0.71
Unsupported	24.6 (11.3)	26.0 (18.1)	0.80
Effective decision	27.8 (10.4)	28.1 (16.3)	0.95
Total score	28.4 (10.3)	29.3 (16.1)	0.87

**Fig. 2:**
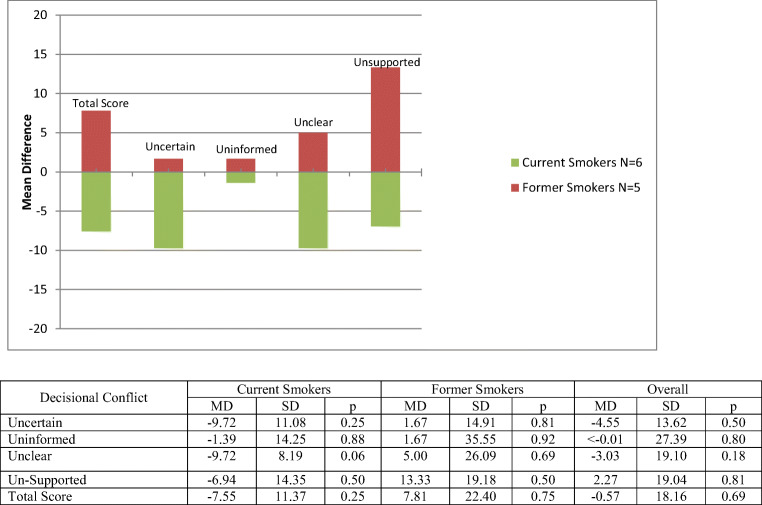
Change in decisional conflict measures according to smoking status

Results on change in decisional conflict are very limited given that only 11 participants completed the follow-up assessment. Findings across all subscales were not significant. Among those 11 individuals, 6 were current smokers and 5 were former smokers. Changes in mean scores between baseline and follow-up for the decisional conflict scale and subscales (uncertain, uninformed, unclear, and unsupported) between current and former smokers appeared to move in opposite directions. Across all subscales, current smokers showed decreased decisional conflict while, conversely, former smokers’ decisional conflict increased (Fig. 2).

Table 3 presents lung cancer screening according to DCP preference score for smokers and former smokers. Current smokers with a higher preference score (favored screening) were significantly more likely to screen (*p* = 0.03) compared with those who had a lower preference score (did not favor screening). This association was not statistically significant among former smokers.

**Table 3:** Preference score and screening

	Screen (*N* = 9)	Did not screen (*N* = 11)	*p* value
Current smoker			
Mean (SD)	0.678 (0.117)	0.456 (0.142)	0.03
Median	0.651	0.531	0.01
*N* = 11	*N* = 6	*N* = 5	
Former smoker			
Mean (SD)	0.587 (0.099)	0.464 (0.197)	0.39
Median	0.642	0.502	0.37
*N* = 9	*N* = 3	*N* = 6	

In data not shown, screening rates were shown to be highest among those participants who completed the DCP intervention (9/20), compared with both those who agreed to participate but did not complete the study (0/8) and compared with individuals who were aware of their eligible status because of our study, but chose not to enroll (3/26).

## Discussion

Given the very low national rate of lung cancer screening, there is a critical need to develop and implement effective strategies which identify and engage eligible patients in a coordinated program of lung cancer screening. SDM must be integral to LCS for the pragmatic purpose of reimbursement requirements but, most importantly, for the best interest of the patient. To our knowledge, this is the first report of a non-persuasive SDM intervention which also increases the rate of lung cancer screening. Models such as this are needed in order to realize the full benefit of lung cancer screening in real-world clinical care. The early literature suggests that SDM in lung cancer screening when incorporated into the usual care process of the patient-physician dyad is probably of low quality [9]. In contrast, our intervention supports patient education and provides the opportunity for the patient to become aware their own values and beliefs related to lung cancer screening while maintaining the patient-physician relationship. Importantly, this intervention allows the SDM process to occur outside of the time-pressured “black box” of the face-to-face physician visit. Our intervention builds upon the patient-physician relationship, a known potent predictor of cancer screening, while minimizing disruption to real-world clinical practice. Of note, the CMS guidelines require a visit with a designated health care provider, e.g., physician, physician assistant, or advanced nurse practitioner, and it is generally interpreted that this visit should be face-to-face. If we had not partnered with primary care and the LHSP program, our intervention would not have met CMS requirements. The alteration of the CMS guidelines would offer the opportunity to use an approach such as ours for outreach to a general population.

Case finding, identification of eligible participants for lung cancer screening, from primary care EMR data was very difficult but comparable with other studies in the literature [5]. This difficulty offers insight into national low rates of LDCT. The eligible population is hard to find. One likely reason is that the number of inclusion/exclusion criteria associated with LCS is just not simple. In addition, the stigma associated with smoking (heavy smoking over many years) could have caused this population to become generally less engaged in the health care system. Nonetheless, it seems reasonable to assume that the population, if reached and made aware, would choose to screen at a higher rate of than the current estimates of 3%. Our case finding challenges highlight the need for intense educational outreach to make the eligible population aware of the opportunity for screening. If a patient does not screen because he/she is unaware of their own eligibility, this is not truly a choice. Further research should allow for enhanced outreach efforts to identify this eligible population, including adequate staffing as well as alternative strategies such as web-based, text messaging, use of patient portals, and engaging with community partners such as quit lines.

Women appeared to be less likely to complete low-dose CT scanning and further research is needed to understand why. Women in the age group for lung cancer screening have a high burden of preventive care including osteoporosis screening, breast cancer screening, cervical cancer screening, and colorectal cancer screening, and thus, women may be reticent to add more to the list. Also, perhaps, as in colorectal cancer, women may not view lung cancer as a “woman’s disease.” Deliberate marketing strategies aimed at women should be strongly considered to prevent disparity.

Current smokers were less likely to complete the DCP. Negative stigma about smoking could make patients who smoke reluctant to engage in an intervention especially if the patient anticipates an unwelcome focus on smoking cessation. Effective engagement strategies which address smoking cessation in a manner that is acceptable to reluctant patients are crucial to the success of lung cancer screening.

A preference score for screening was associated with screening in current smokers but not in former smokers. Further study in a larger population is needed to validate these findings but there may be important differences in screening behavior in these two groups and interventions may need to be tailored accordingly.

A limitation of this study was the measurement of change in decisional conflict. Only 11 participants completed both baseline and follow-up decisional conflict surveys. There was a trend in our data indicating different responses in smokers and former smokers related to decision conflict. This alerts us to the need for better tools to measure decision quality in LCS and the likelihood that not all tools work equally well in all subgroups. Intermediate outcomes such as documentation of whether SDM took place with the physician post-intervention were not captured and would be important in future studies. Also, follow-up data on decisional conflict was collected at 30 days, around the average time of LDCT completion, and the act of completing a LDCT is a potential confounding variable. Future work would need to account for the influence of actually getting the LDCT on the individual’s sense of decisional conflict. For example, individuals who completed LDCT and have an abnormal result may have experience more conflict and general dissatisfaction with their decision while those who have a normal result may feel less conflicted in response to the news.

Participation in the follow-up phone call (post decisional conflict survey) was challenging. We attempted to mail a paper copy of the survey to those individuals who were not reached by phone with minimal response. Our qualitative experience suggests that people were disinterested in further contact after the first phone call and phone-based intervention. Strategies in future work will include reducing the duration of the first phone call to minimize any negative expectations regarding time required for follow-up call, offering email options, and offering a patient incentive for completion.

The small sample size is the major limitation of this study. However, this study was initially conceived as a feasibility study. Future research studies should enhance recruitment, increase the study sample size, and include formal comparison strategies. Future studies should also include cost analysis in order to address the sustainability of this type of SDM model.

## Conclusion

The integrated DCP, a telephone-based primary care-embedded, decision counselor–administered online DCP tool, promotes lung cancer screening. Equally important, the integrated DCP intervention is feasible in real-world clinical care and maintains a high level of quality SDM. Best practices for implementing shared decision-making and increasing LDCT in real-world clinical care are needed if the full benefit of lung cancer screening is to be realized.
